# Supplementary material for: Tumor Signature Analysis Implicates Hereditary Cancer Genes in Endometrial Cancer Development
Source: Cancers (Basel). 2021 Apr 7;13(8):1762. doi: 10.3390/cancers13081762 (PMC8067736; doi:10.3390/cancers13081762)
Supplement: Supplementary file 1 [file cancers-13-01762-s001.zip › Kondrashova_etal_Supp_Info.pdf]

Supplementary Information:

Tumor signature analysis implicates hereditary cancer genes in endometrial cancer development

Olga Kondrashova[1*], Jannah Shamsani[1*], Tracy A. O'Mara[1], Felicity Newell[1], Amy E. McCart Reed[2], Sunil R Lakhani[2,3], Judy Kirk[4], John V. Pearson[1], Nicola Waddell[1*] and Amanda B. Spurdle[1*]

*These authors contributed equally to the work

Author information

1. Department of Genetics and Computational Biology, QIMR Berghofer Medical Research Institute, Brisbane, Australia
2. UQ Centre for Clinical Research, Faculty of Medicine, The University of Queensland, Brisbane, Australia.
3. Pathology Queensland, Brisbane, Australia.
4. Familial Cancer Service, Crown Princess Mary Cancer Centre, Westmead Hospital, Sydney Medical School, University of Sydney, Centre for Cancer Research, The Westmead Institute for Medical Research, Westmead, NSW, Australia

Supplementary Tables

Table S1: Subset of European TCGA-UCEC cases included in the variant frequency comparison with the gnomAD population of Non-Finnish Europeans. (provided in separate Excel file)

Table S2: Phenotypic characteristics of the familial endometrial cancer cases. (provided in separate Excel file)

Table S3: Summary of sequencing coverage and number of variants identified for each familial endometrial cancer case prior to filtering. (provided in separate Excel file)

Table S4: List of known and purported endometrial cancer risk genes. (provided in separate Excel file)

Table S5: List of pathogenic and likely pathogenic variants identified in the genes of interest in gnomAD (non-Finnish Europeans) and TCGA-UCEC cases. (provided in separate Excel file)

Supplementary Figures

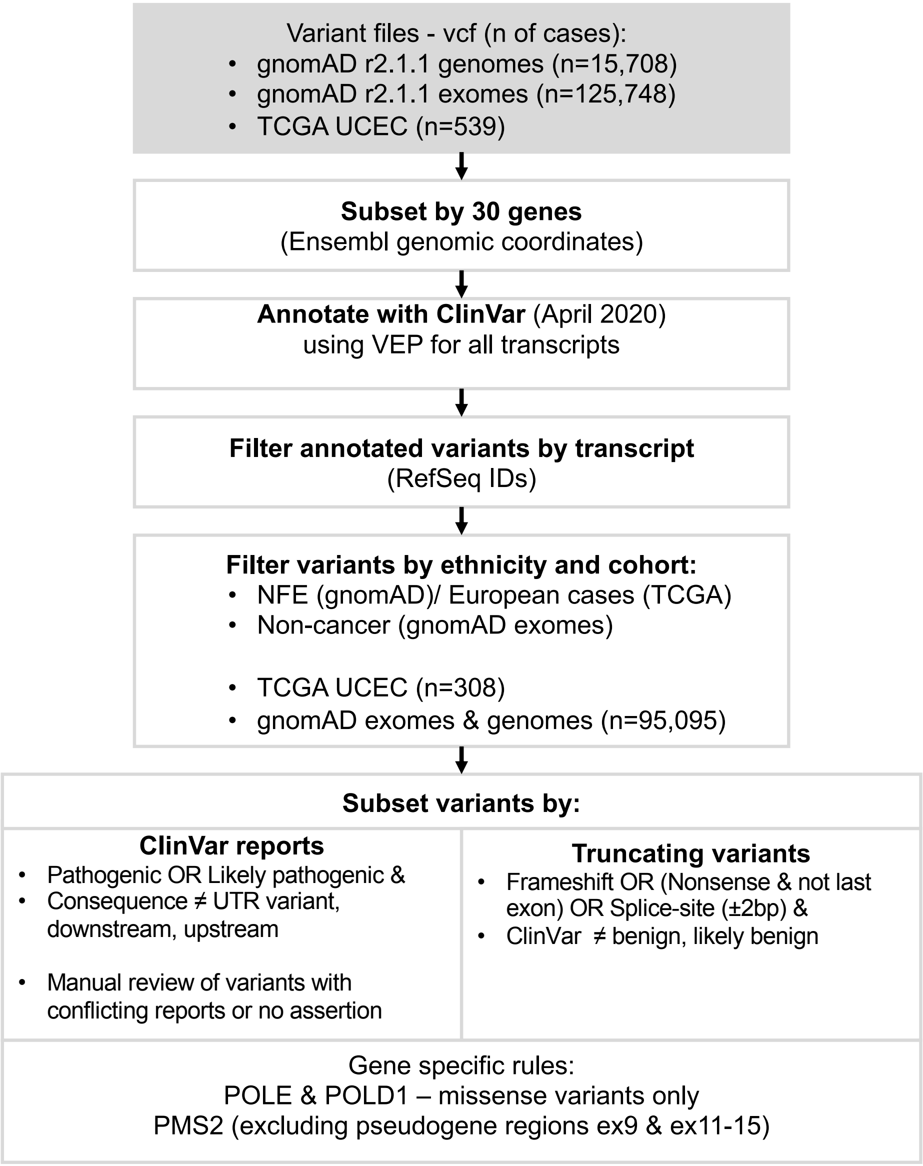

Figure S1: Variant filtering workflow used for germline pathogenic and likely pathogenic variants included in this study for TCGA-UCEC and gnomAD datasets. NFE – Non-Finnish Europeans; VEP – Variant Effect Predictor; UTR – Untranslated region.

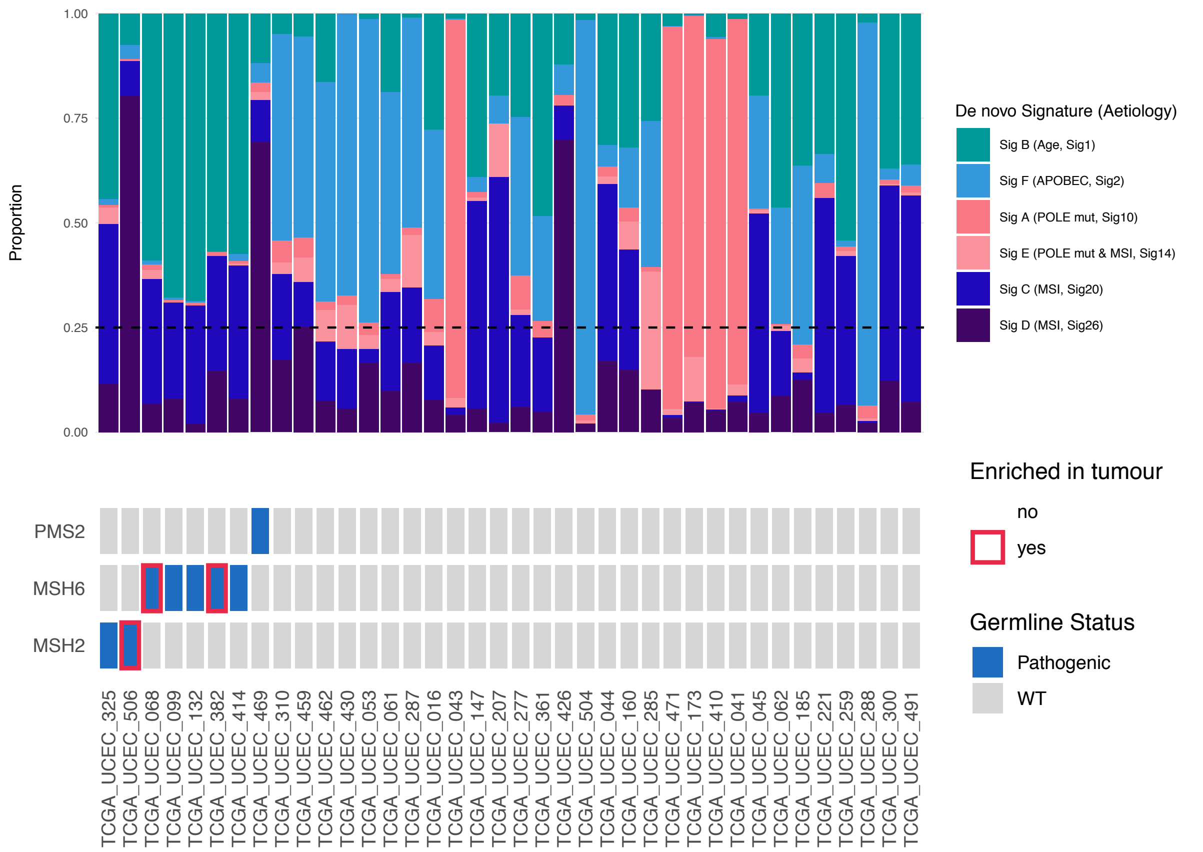

Figure S2: ***De novo*** **mutational signature analysis in the TCGA-UCEC cases with pathogenic or likely pathogenic variants.** Germline variant status for MMR pathway genes is shown. MMR – mismatch repair; MSI – microsatellite instability; WT – wild-type.
